# Meat and dairy products intake is associated with gastric cancer: Case–control study findings

**DOI:** 10.1002/fsn3.3364

**Published:** 2023-04-12

**Authors:** Reema F. Tayyem, Hala Nawaiseh, Narmeen Al‐Awwad, Tareq Al‐Jaberi, Ahmad Hushki, Sabika Allehdan

**Affiliations:** ^1^ Department of Human Nutrition, College of Health Sciences Qatar University Doha Qatar; ^2^ Department of Nutrition and Food Technology, Faculty of Agriculture University of Jordan Amman Jordan; ^3^ Department of Clinical Nutrition and Dietetics, Faculty of Applied Health Sciences The Hashemite University Zarqa Jordan; ^4^ Department of General and Pediatric Surgery, Faculty of Medicine Jordan University of Science and Technology Irbid Jordan; ^5^ Gastroenterology Division King Hussein Cancer Center Amman Jordan; ^6^ Department of Biology, College of Science University of Bahrain Zallaq Bahrain

**Keywords:** case–control study, dairy products, eggs, gastric cancer, meats

## Abstract

Countries experiencing a significant shift toward Western lifestyle are facing an increased risk of gastric cancer (GC). While many studies have explored the link between diet and GC, the role of meat and dairy consumption remains uncertain. To delve deeper into this association, we conducted a case–control study in Jordan involving 173 GC cases and 314 controls, matched by age and marital status. Using a validated food frequency questionnaire, we assessed the intake of different dairy and meat products. The adjusted odds ratios and corresponding 95% confidence intervals indicated a strong correlation between high intake of red meat, milk, and buttermilk and GC. Our multinomial logistic regression analysis revealed that daily consumption of red meat (≥1 serving/day; OR, 3.34 [95% CI 1.85–6.03, *p* value of trend <.001], ≥1 serving/day) and weekly intake of milk (2–3 servings/week; OR, 2.04 [95% CI 1.14–3.64, *p* value of trend = .041]) and buttermilk (2–3 servings/week; OR, 2.07 [95% CI 1.12–3.83, *p* value of trend = .018], per 2–3 servings/week) were significantly associated with an increased risk of GC. Furthermore, consuming cooked eggs daily (OR = 2.44, 95% CI 1.24–4.79, *p* trend <.001) or weekly (OR, 3.34, 95% CI 1.58–7.10, *p* value of trend <.001) was also associated with an increased risk of GC. These findings highlight the potential health risks associated with excessive meat and some dairy products consumption and suggest that a balanced intake of these products, along with eggs, may help prevent GC.

## Graphical abstract

The findings of this study suggest that high consumption of some dairy products, meat, and eggs may adversely affect health and increase the risk of developing GC. Consuming an adequate amount of dairy products, meats, and eggs could be a preventive measure to reduce the risk of GC.

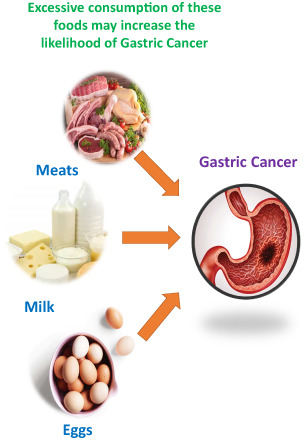

## 1 INTRODUCTION

Gastric cancer (GC) presents the fourth most common cancer in men and the seventh most common cancer in women. There were more than 1 million new cases of GC and 768,793 deaths worldwide in 2020 (WCRF‐International, 2022). The increasing trend in the prevalence of GC and its high mortality rate urgently required finding innovative strategies to prevent this disease (Zhao et al., 2017). Identification of risk factors could have a remarkable effect on reducing GC morbidity and mortality (Larsson et al., 2006). Growing evidence from different researches indicates that *Helicobacter pylori* infection, genetic instability, and environmental and behavioral variables, particularly diet and food intake, all contribute to the development of GC (Tayyem et al., 2022). Several studies have been conducted on the relationships between various dietary patterns and ingredients and GC, in light of the hypothesis that diet plays a significant role in the genesis of these malignancies (Tayyem et al., 2022; Zamani et al., 2013).

High‐quality evidence investigated that red meat, particularly processed meat, was associated with an increased risk of digestive system malignancies, including GC (Larsson et al., 2006; Zamani et al., 2013; Zhao et al., 2017). However, several studies on this association showed controversial results (Larsson et al., 2006; Zamani et al., 2013; Zhao et al., 2017). Along with high amounts of salt, processed meat frequently includes carcinogenic N‐nitroso compounds (NOCs) and polycyclic aromatic hydrocarbons (Larsson et al., 2006). Additionally, heme iron content which might cause oxidative damage and the high energy density of meat might also contribute to carcinogenesis (Zamani et al., 2013; Zhao et al., 2017). Zamani et al. (2013) showed that red meat consumption was positively associated with GC risk (OR = 1.87, 95% CI 1.01–3.47, *p* trend = .07; Zamani et al., 2013). However, an inverse association between white meat consumption (OR = 0.36, 95% CI 0.19–0.68, *p* trend = .005) and the risk of GC was detected (Zamani et al., 2013). Few studies have investigated the role of dairy products and the risk of GC (Navarro Silvera et al., 2008). A meta‐analysis reported that no significant correlation exists between the consumption of dairy products and the risk of GC (Tian et al., 2014). Regarding egg consumption and GC risk, a study conducted by Flores‐Luna et al. (2020) revealed that egg intake (OR = 1.7 95% CI 1.1–2.6; *p* = .021) was associated with GC (Flores‐Luna et al., 2020). Two ecological studies in high‐risk countries, China and Brazil, reported geographical positive correlations between GC mortality and egg consumption at a population level (Kneller et al., 1992; Sichieri et al., 1996).

Therefore, considering the substantial prevalence of GC worldwide and the controversial evidence, this research aimed to evaluate the association between the consumption of dairy products; red, white, and processed meats; and eggs and the odds of GC in Jordan.

## 2 METHODS

### 2.1 Study design and participants

The present study was a case–control study and was conducted from March 2015 to August 2018 in four major hospitals which have an oncology unit in Jordan. The study was approved by the IRB Committee of the King Hussein Cancer Center (IRB No. 15 KHCC 03, Amman, Jordan) and the other hospitals.

A total of 487 participants including 173 GC cases and 314 controls (selected from the community) were enrolled in this study. The controls were matched to the cases based on age and marital status. The ratio of cases to controls in this study was 1:1. Inclusion criteria were Jordanian aged 18 years or above, able to talk, and free of diet‐related diseases, GC confirmed for the cases, being free of GC for the controls. Patients who were critically ill, unable to talk, on special diets, and diagnosed with neoplastic condition for more than 6 months, GC as a second cancer or liver, gastrointestinal or renal diseases were all excluded. All participants were asked to give a signed written informed consent.

### 2.2 Data collection

Structured questionnaires were used to collect information regarding sociodemographic and health characteristics, physical activity, and dietary intake. These questionnaires were completed by trained interviewers for both cases and controls. Anthropometric measurements were taken by trained nutritionists as described by Lee and Nieman (2013). Body mass index (BMI) was then calculated (Lee & Nieman, 2013).

#### 2.2.1 Anthropometric measurements

Participants' current and pre‐diagnosis body weight and height were measured using standardized techniques and calibrated tools by a trained dietician (Lee & Nieman, 1996). BMI was computed as the ratio of weight (kg) to height squared (m; Lee & Nieman, 1996) and classified according to World Health Organization guidelines (Diet, 2003). However, the pre‐diagnosis body weight before diagnosis was self‐reported from the cases and controls.

### 2.3 Physical activity questionnaire

In‐person interview was used to collect data about the physical activity level of participants. A validated 7‐day physical activity recall was completed for each participant to estimate the level of physical activity (Sallis et al., 1985; Washburn et al., 2003). Metabolic equivalents (METs) and the total physical activity MET‐minute per week were determined according to Washburn et al. (2003). Participants were asked to recall the frequency, intensity, duration, type, and time spent doing physical activity over a 7‐day period. In addition to that, sleeping hours were also reported by the participants and were used along with the reported physical activity level to be converted into MET. Sleeping was assigned a value of 1.0 MET, light activity a value of 1.5 METs, moderate activity a value of 4.0 METs, and very hard activity a value of 7.0 METs or greater, according to the scoring instructions (Sallis et al., 1985).

### 2.4 Dietary intake assessment

The consumption of dairy products, eggs, and meats was assessed using a validated food frequency questionnaire (FFQ; Tayyem et al., 2014). Participants were asked about their dairy product, eggs, and meat intake over the past 12 months. The FFQ consists of 13 items of meats and processed meats, fish, and eggs and eight items of dairy products. For each type of meats and dairy products, the participants were asked whether they had consumed or not and to recall how frequently, on average, during the past year they had consumed one standard serving of a specific food item in 10 classes (1–6 times/year, 7–11 times/year, 1 time/month, 2–3 times/month, 1 time/week, 2 times/week, 3–4 times/week, 5–6 times/week, 1 day, 2 or more/day). The portion sizes of each food item were estimated according to commonly used portion sizes into three categories (small, medium, or large). Standard measuring tools (e.g., cup, tablespoon, teaspoon, and glass) and food models were used to assess the consumed portion size of food item of dairy products and meats exactly. Knowing the frequency of consumption and the specified serving size for each food item, the average daily amount of each food item consumed of the dairy products, meats, fish, and eggs was then calculated for each participant.

### 2.5 Statistical analysis

Descriptive analyses were performed to examine differences between participants based on their consumption frequencies. Normally distributed continuous variables were reported as mean ± standard error of the mean, and the categorical variables were reported as frequency and percentage. The normality of continuous variables was assessed by the Shapiro test. One‐way ANOVA was used to compare differences in the mean of continuous variables. Pearson chi‐square was used to find differences in categorical variables. Median and interquartile were computed for representing the intake of dairy products and meats as number of consumed servings per week. Mann–Whitney *U* test was used to detect differences in intakes of selected food items of dairy products and meats between GC cases and controls. Multinomial logistic regression was used to calculate odds ratios (ORs) and their corresponding 95% confidence intervals (95% CIs) according to different categories of consumption of dairy products and meats during a week. The reference group was the category with the lowest intake of consumption (≤1 serving per week). Several variables were selected as potential confounders (caloric intake, age, gender, marital status, education level, body weight status, smoking, period of smoking, family history of GC, and physical activity level) based on mentioned risk factors for GC in some studies (Al‐Awwad et al., 2021; Nomura et al., 2003; Toorang et al., 2021). *p* value for trend was calculated using linear logistic regression. Statistical analysis was conducted using SPSS version 28, and a value of *p* < .05 was considered statistical.

## 3 RESULTS

A total of 487 participants (298 male and 189 female) from Jordan were included in this study. Table 1 shows the distribution of sociodemographic and health characteristic of the study participants by the frequency of consumption of dairy products and meats. No significant differences were detected in age, gender, body weight status, marital status, educational level, occupational status, smoking, personal history of chronic diseases (diabetes, cardiovascular diseases, hypertension, and arthritis), and family history of GC when the participants were categorized according to number of dairy products servings. Frequency of females (80.4%) who consumed dairy products on daily basis was more significant than that of males (67.4%; *p* = .01). Majority of GC cases (96. 5%) consumed dairy products daily (*p* < .001). Total caloric intake and physical activity level were statistically significant among different levels of dairy products consumption. On contrast, significant differences were detected in age, height, current BMI, tobacco use, total energy intake, physical activity level, and occupational status among participants, which classified based on number of meats servings. The proportion of GC cases who consumed meats on daily basis was significantly higher than that of controls (*p* < .001; Table 1).

**TABLE 1:** Sociodemographic and health characteristics of the study participants by frequency of dairy products and meats consumption.

Variable	Dairy products (*N* = 487)					Meats (*N* = 487)				
	Rarely (*n* = 2)	Monthly (*n* = 31)	Weekly (*n* = 101)	Daily (*n* = 353)	*p*‐value*	Rarely (*n* = 2)	Monthly (*n* = 94)	Weekly (*n* = 278)	Daily (*n* = 113)	*p*‐value*
Age (years)	50.0 ± 5.0	51.1 ± 2.3	56.7 ± 1.2	53.5 ± 0.67	.058	56.0 ± 9.0	52.56 ± 1.4	55.5 ± 0.74	51.6 ± 1.1	.012
Previous weight (kg)	68.0 ± 11.0	73.7 ± 3.3	83.1 ± 2.3	82.0 ± 1.1	.121	87.5 ± 17.5	82.1 ± 1.3	81.7 ± 2.1	82.3 ± 1.7	.766
Current weight (kg)	69.5 ± 9.5	76.6 ± 2.4	76.6 ± 2.3	76.0 ± 2.4	.054	78 ± 8.0	78.9 ± 1.7	78.3 ± 1.1	73.6 ± 1.4	.176
Height (cm)	164.0 ± 4.0	167.6 ± 8.5	168.4 ± 7.8	167.9 ± 8.9	.782	171.0 ± 4.0	164.0 ± 4.0	164.0 ± 4.0	164.0 ± 4.0	.009
Previous BMI (kg/m^₂^)	25.1 ± 2.9	26.5 ± 1.2	29.4 ± 0.81	29.0 ± 0.37	.089	27.7 ± 0.56	27.7 ± 0.56	27.7 ± 0.56	27.7 ± 0.56	.860
Current BMI (kg/m^₂^)	25.7 ± 2.3	27.5 ± 0.28	29.2 ± 0.76	27.0 ± 0.31	.068	26.6 ± 1.5	29.0 ± 0.60	27.7 ± 040	25.7 ± 0.48	.001
Total caloric intake (kcal/day)	2296.6 ± 325.7	1914.6 ± 97.4	2180.4 ± 64.6	3095.5 ± 136.6	<.001	1637.6 ± 661.5	2157.0 ± 64.8	2749.0 ± 168.7	3598.5 ± 97.7	<.001
Physical activity (MET‐min/week)	10260.0 ± 942.3	3434.9 ± 635.1	3179.1 ± 280.0	2797.9 ± 140.2	.001	1140.0 ± 300.0	3175.9 ± 272.7	3008.2 ± 176.4	2644.8 ± 258.8	.043

	*n* (%)					*n* (%)				
Gender										
Male (*n* = 298)	1 (0.33%)	20 (6.7%)	76 (25.5%)	201 (67.4%)	.010	1 (0.33)	50 (16.8%)	169 (56.7%)	78 (26.2%)	.134
Female (*n* = 189)	1 (0.53%)	11 (5.8%)	25 (13.23)	152 (80.4%)		1 (0.53%)	44 (23.3%)	109 (57.7%)	35 (18.5%)	
GC cases (*n* = 173)	0	0	6 (3.4%)	167 (96.5%)	<.001	1 (0.58%)	8 (4.6%)	94 (54.3%)	70 (40.5%)	<.001
Control(*n* = 314)	2 (0.64%)	31 (9.9%)	95 (30.3%)	186 (59.2%)		1 (0.32%)	86 (27.4%)	184 (58.6%)	43 (13.7%)	
Marital status										
Married (*n* = 421)	2 (0.48%)	26 (6.2%)	94 (22.3%)	299 (71.0%)	.395	2 (0.48%)	80 (19.0%)	243 (57.7%)	96 (22.8%)	.964
Single (*n* = 28)	0	4 (14.3%)	2 (7.1%)	22 (78.6%)		0	7 (25.0%)	14 (50.0%)	7 (25.0%)	
Divorced (*n* = 10)	0	0	2 (20.0%)	8 (80.0%)		0	2 (20.0%)	4 (40.0%)	4 (40.0%)	
Widowed (*n* = 28)	0	1 (3.5%)	3 (10.7%)	24 (85.7%)		0	5 (17.9%)	17 (60.7%)	6 (21.4%)	
Educational level										
Less than high school diploma (*n* = 279)	1 (0.36%)	19 (6.8%)	56 (20.1%)	202 (72.4%)	.944	1 (0.36%)	60 (21.5%)	164 (58.8%)	54 (19.4%)	.09
High school diploma and above (*n* = 208)	1 (0.48%)	12 (5.8%)	45 (21.6%)	150 (72.1%)		1 (0.48%)	34 (16.3%)	114 (54.8%)	59 (28.4%)	
Working (*n* = 235)	1 (0.43%)	14 (6.0%)	52 (22.1%)	167 (71.1%)	.844	1 (0.43%)	41 (17.4%)	123 (52.3%)	70 (29.8%)	.027
History of Chronic Diseases (*n* = 233)	2 (0.86%)	13 (5.6%)	44 (18.9%)	174 (74.7%)	.579	1 (0.43%)	43 (18.5%)	147 (63.1%)	42 (18.0%)	.161
Smoking (*n* = 155)	1 (0.64%)	13 (8.4%)	33 (21.3%)	108 (69.7%)	.587	1 (0.64%)	35 (22.6%)	77 (49.7%)	42 (27.1%)	.106
Family history of GC (*n* = 22)	0	2 (9.1%)	0	20 (90.9%)	.101	0	4 (18.2%)	13 (59.1)	5 (22.7%)	.988

Abbreviation: MET, metabolic equivalent.

* *p* values calculated by Sample *t*‐test for continuous variables and *Pearson X* ^*2*^ for categorical variables. *p* value <.05 was considered statistically significant.

Table 2 shows the median weekly intakes and the 25th and 75th percentile of selected food items of dairy products and meats. GC cases showed a significant higher consumption of milk, yogurt, white cheese, processed cheese, cooked red meats, cooked chicken, and cooked eggs than controls (*p* < .05). The intake of cooked yogurt, drained yogurt (Labneh), ice cream, grilled meat, cooked liver, fried and roasted fish, tuna, and processed meats was not statistically significant between cases and controls.

**TABLE 2:** Median weekly intake of dairy products and meats for cases and controls.

Food item (serving size)	Median (25–75th percentile)		*p*‐value*
	Cases servings/week	Control servings/week	
Milk (cup)	1.9 (0.0–8.8)	0.73 (0.0–4.4)	.034
Yogurt (cup)	3.5 (2.0–7.0)	2.7 (1.4–4.7)	.001
Cooked yogurt (Jameed; cup)	0.44 (0.17–0.74)	0.44 (0.17–0.74)	.213
Butter milk (cup)	0.74 (0.17–2.1)	0.44 (0.17–0.98)	.002
Drained yogurt (Labneh; 1/4 cup)	5.1 (2.0–7.0)	5.1 (2.0–7.0)	.333
White cheese (oz)	3.0 (1.5–7.0)	1.5 (0.49–3.5)	<.001
Processed cheese (oz)	1.5 (0.23–3.5)	0.58 (0.07–2.0)	<.001
Ice cream (1/2 cup)	1.1 (0.38–1.9)	0.41 (0.14–1.9)	.271
Cooked red meat (veal; oz)	5.4 (3.2–11.2)	3.2 (1.3–5.4)	<.001
Grilled meat (oz)	0.61 (0.30–1.2)	0.81 (0.25–1.2)	.756
Cooked red meat (lamb; oz)	3.4 (0.81–7.1)	2.0 (0.71–3.4)	<.001
Cooked chicken (oz)	8.1 (3.9–14.0)	7.0 (2.3–12.2)	.001
Cooked liver (oz)	0.55 (0.17–1.4)	0.55 (0.27–1.4)	.492
Fried fish (oz)	1.0 (0.32–2.1)	1.0 (0.49–1.62)	.092
Roasted fish (oz)	0.35 (0.0–1.0)	0.81 (0.0–1.6)	.033
Tuna (oz)	0.69 (0.58–2.5)	0.64 (0.46–1.7)	.142
Cooked egg (egg)	3.5 (1.96–7.0)	2.0 (0.98–4.1)	<.001
Beef mortadella (oz)	0.0 (0.0–0.44)	0.0 (0.0–0.44)	.681
Chicken mortadella/processed turkey (oz)	0.0 (0.0–0.44)	0.0 (0.0–1.1)	.139

* *p* values were calculated by *Mann–Whitney U* test and *p* value <.05 was considered statistically significant.

Table 3 presents crude ORs and 95% CIs for dairy products and meats. The intake of different levels and frequencies of milk, yogurt, white and processed cheeses, cooked veal meat, cooked chicken, and eggs had positive significant associations with the risk of GC. However, the consumption of buttermilk on a weekly basis increased the risk of GC two‐folds (*p* trend <.001). The odd of GC was found to be higher with the daily consumption of white cheese (OR = 4.34, 95% CI 2.48–7.59, *p* trend <.001) and cooked veal meat (OR = 4.05, 95% CI 1.84–8.92, *p* trend <.001).

**TABLE 3:** Crude odds based on the number of dairy products and meats servings consumed among cases and controls.

Food item (serving size)	Crude odd ratios (95% CI)				*p* value of trend
	≤1 serving per week^a^	2–3 servings per week	4–6 servings per week	≥1 serving per day	
Milk (cup)	1	1.74(1.05–2.87)	2.18(1.07–4.43)	1.84(1.15–2.94)	.005
No. of cases/control	73/180	37/52	17/21	46/61	
Yogurt (cup)	1	1.82(1.00–3.1)	1.47(0.66–3.31)	2.70(1.47–4.99)	.004
No. of cases/control	23/73	91/159	13/28	46/54	
Cooked yogurt (Jameed; cup)	1	0.67(0.26–1.74)	–	–	.270
No. of cases/control	167/297	6/16	0/0	0/1	
Drained Yogurt (Labneh; 1/4 cup)	1	1.68(0.95–2.97)	0.75(0.34–1.66)	1.29(0.75–2.21)	.976
No. of cases/control	26/60	59/81	12/37	76/136	
Buttermilk (cup)	1	2.30(1.44–3.65)	2.04(1.00–4.16)	1.44(0.46–4.49)	<.001
No. of cases/control	105/241	47/47	16/18	5/8	
White cheese (oz)	1	2.96(1.81–4.82)	3.64(1.87–7.07)	4.34(2.48–7.59)	<.001
No. of cases/control	34/142	69/99	24/28	46/45	
Processed cheese(oz)	1	1.78(1.14–2.76)	2.76(1.21–6.29)	3.56(1.90–6.68)	<.001
No. of cases/control	81/209	51/73	13/12	28/20	
Ice cream (1/2 cup_	1	1.61(1.05–2.45)	1.52(0.85–2.73)	0.64(0.28–1.47)	.684
No. of cases/control	79/170	62/83	24/34	8/27	
Cooked red meat (veal; oz)	1	1.77(0.83–3.77)	2.12(0.97–4.67)	4.05(1.84–8.92)	<.001
No. of cases/control	10/39	65/143	43/79	55/53	
Cooked red meet (lamb; oz)	1	0.91(0.59–1.43)	1.85(0.48–7.14)	3.24(1.95–5.38)	<.001
No. of cases/control	3/17	16/50	36/83	118/164	
Grilled red meat (oz)	1	1.07(0.71–1.61)	0.92(0.34–2.53)	1.03(0.34–3.14)	.909
No. of cases/control	110/203	52/90	6/12	5/9	
Cooked Chicken (oz)	1	2.32(1.03–5.22)	5.93(2.28–15.40)	3.50(1.65–7.43)	<.001
No. of cases/control	9/48	37/85	20/18	107/163	
Cooked liver (oz)	1	1.05(0.68–1.62)	2.45(0.54–11.12)	0.55(0.15–2.04)	.906
No. of cases/control	122/224	44/77	4/3	3/10	
Cooked fish (oz)	1	0.57(0.37–0.87)	0.62(0.34–1.11)	0.71(0.30–1.71)	.091
No. of cases/control	66/82	75/167	23/49	9/16	
Tuna (oz)	1	1.0(0.67–1.49)	1.61(0.83–3.12)	0.27(0.03–2.19)	.773
No. of cases/control	192/171	61/114	19/22	1/7	
Cooked egg (egg)	1	1.25(0.75–2.08)	2.57(1.37–4.81)	2.34(1.36–4.02)	<.001
No. of cases/control	32/90	55/129	32/35	54/65	
Beef mortadella (oz)	1	0.44(0.23–0.83)	0.78(0.29–2.08)	2.94(0.85–10.22)	.814
No. of cases/control	147/247	13/50	6/13	7/4	
Chicken mortadella/ processed turkey (oz)	1	0.56(0.31–1.02)	0.67(0.25–1.85)	0.73(0.19–2.79)	.071
No. of cases/control	139/220	22/69	8/16	4/9	

*Note*: *p* value <.05 was considered statistically significant.

^a^ Reference group. The control group was considered the reference group for analysis.

Table 4 shows that the daily consumption of white cheese (OR = 4.18, 95% CI 1.97–8.86, *p* trend <.001), processed cheese (OR = 3.25, 95% CI 1.57–6.75, *p* trend <.001), cooked red meat (veal; OR = 4.27, 95% CI 1.62–11.2, *p* trend = .011), cooked red meat (lamb; OR = 3.34, 95% CI 1.85–6.03, *p* trend <.001), cooked chicken (OR = 3.00, 95% CI 1.29–7.02, *p* trend = .003), and cooked eggs (OR = 2.44, 95% CI 1.24–4.79, *p* trend <.001) was positively associated with the incidence of GC. In general, as the number of white cheeses, processed cheese, and red meats servings increased to be 1 or more serving(s) per day, the incidence of GC significantly increased. Significant associations were found for weekly consumption of milk (OR, 2.04 [95% CI 1.14–3.64, *p* value of trend = .041], per 2–3 servings/week) and buttermilk (OR, 2.07 [95% CI 1.12–3.83, *p* value of trend = .018], per 2–3 servings/week), white cheese (OR, 2.94 [95% CI 1.50–3.63, *p* value of trend <.001], per 2–3 servings/week), processed cheese (OR, 1.85 [95% CI 1.11–3.08, *p* value of trend <.001], per 2–3 servings/week), cooked chicken (OR, 5.31 [95% CI 1.81–15.54, *p* value of trend = .003], per 4–6 servings/week), and cooked eggs (OR, 3.34 [95% CI 1.58–7.10, *p* value of trend <.001], per 4–6 servings/week). The results show that the consumption of yogurt, drained yogurt (Labaneh), cooked yogurt, ice cream, grilled meat, cooked fish, tuna, cooked liver, and processed meats had no effect on GC risk.

**TABLE 4:** Adjusted odds based on the number of dairy products and meats servings consumed among cases and controls.

Food item (serving size)	AOR (95% CI)^a^				*p*‐value of trend
	≤1 serving per week^b^	2–3 servings per week	4–6 servings per week	≥1 serving per day	
Milk (cup)	1	2.04(1.14–3.64)	1.75(0.77–3.90)	1.66(0.94–2.95)	.041
No. of cases/control	73/180	37/52	17/21	46/61	
Yogurt (cup)	1	1.74(0.95–3.19)	1.12(0.44–2.85)	1.77(0.86–3.66)	.109
No. of cases/control	23/73	91/159	13/28	46/54	
Cooked yogurt (Jameed; cup)	1	1.12(0.57–2.22)	‐	‐	.129
No. of cases/control	167/297	6/16	0/0	0/1	
Drained Yogurt (Labneh; 1/4 cup)	1	0.91(0.45–1.88)	1.02(0.40–2.64)	1.13(0.53–2.40)	.832
No. of cases/control	26/60	59/81	12/37	76/136	
Buttermilk (cup)	1	2.07(1.12–3.83)	1.89(0.75–4.76)	1.51(0.28–8.16)	.018
No. of cases/control	105/241	47/47	16/18	5/8	
White cheese (oz)	1	2.94(1.5–5.63)	2.69(1.11–6.5)	4.18(1.97–8.86)	<.001
No. of cases/control	34/142	69/99	24/28	46/45	
Processed cheese (oz)	1	1.85(1.11–3.08)	2.19(0.87–5.54)	3.25(1.57–6.75)	<.001
No. of cases/control	81/209	51/73	13/12	28/20	
Ice cream (1/2 cup_	1	1.51(0.92–2.48)	1.30(0.63–2.70)	0.64(0.24–1.69)	.741
No. of cases/control	79/170	62/83	24/34	8/27	
Cooked red meat (veal; oz)	1	2.18(0.86–5.54)	2.43(0.93–6.36)	4.27(1.62–11.2)	.011
No. of cases/control	10/39	65/143	43/79	55/53	
Cooked red meet (lamb; oz)	1	1.00(0.60–1.67)	1.36(0.23–8.12)	3.34(1.85–6.03)	<.001
No. of cases/control	3/17	16/50	36/83	118/164	
Grilled red meat (oz)	1	1.04(0.63–1.74)	1.48(0.39–5.56)	1.37(0.41–4.62)	.910
No. of cases/control	110/203	52/90	6/12	5/9	
Cooked Chicken (oz)	1	1.61(0.65–4.01)	5.31(1.81–15.54)	3.00(1.29–7.02)	.003
No. of cases/control	9/48	37/85	20/18	107/163	
Cooked liver (oz)	1	0.92(0.55–1.53)	1.66(0.27–10.35)	0.77(0.19–3.05)	.651
No. of cases/control	122/224	44/77	4/3	3/10	
Cooked fish (oz)	1	0.66(0.40–1.08)	0.57(0.28–1.14)	0.67(0.24–1.89)	.080
No. of cases/control	66/82	75/167	23/49	9/16	
Tuna (oz)	1	0.96(0.60–1.55)	1.59(0.72–3.48)	0.35(0.04–3.21)	.566
No. of cases/control	192/171	61/114	19/22	1/7	
Cooked eggs (egg)	1	1.43(0.78–2.64)	3.34(1.58–7.10)	2.44(1.24–4.79)	<.001
No. of cases/control	32/90	55/129	32/35	54/65	
Beef mortadella (oz)	1	0.38(0.18–0.80)	0.62(0.20–1.97)	1.90(0.50–7.31)	.257
No. of cases/control	147/247	13/50	6/13	7/4	
Chicken mortadella/ processed turkey (oz)	1	0.56(0.31–1.02)	0.67(0.25–1.85)	0.73(0.19–2.79)	.112
No. of cases/control	139/220	22/69	8/16	4/9	

*Note*: *p* value <.05 was considered statistically significant.

^a^ Adjusted for caloric intake, age, gender, marital status, education level, body weight status, smoking, period of smoking, family history of GC, and physical activity level. The control group was considered the reference group for analysis.

^b^ Reference group. The control group was considered the reference group for analysis.

## 4 DISCUSSION

The current study aimed to assess the association between dairy products, red, white, and processed meats and egg consumption and the risk of GC in Jordan. The findings of this study showed that patients who had a higher consumption of cooked red meat (veal and lamb) and poultry were more likely to suffer from GC. This finding is consistent with the meta‐analysis from cohort and case–control studies which indicated that individuals with a higher red meat intake had a 41% risk to develop GC compared with individuals with the lowest red meat consumption (relative risk (RR) = 1.41; Kim et al., 2019). Moreover, the current findings are also consistent with data from a case–control study to assess the dietary factor associated with GC, and it had been observed that the intake of charcoal‐grilled beef significantly increased the risk of GC (OR = 7.7, *p* value <.001; Kim et al., 2002). The current data are also consistent with those reported that the high intake of total meat (serving/week) was significantly associated with increased risk of GC for the highest quartile (≥10.95 serving/week) vs. the lowest quartile (≤3.94 serving/week; OR = 1.6; *p* value <.05; Hu et al., 2008). The dose–response analysis of four cohort studies and 13 case–control studies have indicated that each 100 g/day increase in red meat consumption among the individuals was associated with a 26% increased risk of GC (RR, 1.2; Kim et al., 2019). In addition, data from a case–control study in Uruguay showed that higher risk of GC was associated with high consumption stewed meat (OR, 2.02; 95% CI 1.36–2.99). However, red meat, white meat (poultry and fish), total meat, and dairy foods were not associated with risk of GC (De Stefani et al., 2004). Inconsistent with the current result, a study by conducted by Hu et al. (2008) reported that a high intake of poultry was associated with a 30% reduction in risk of GC in men (for the highest vs. the lowest quartile; OR = 0.7; Hu et al., 2008). The “mixed pattern” which is defined as relatively high loadings of red meat, processed meat, eggs, and pulses was not associated with the risk of GC (crude OR for the highest load, 0.99; *p* value = .98; Hu et al., 2008).

However, after adjusting for total energy intake, the highest load was strongly protective (OR, 0.59; *p* value = .01; De Stefani et al., 2004). Several studies showed a statistically significant elevated risk concerning fresh red meat (Correa et al., 1985) or processed meat intake and the risk of GC (Takezaki et al., 2001). The frequency score for beef intake (OR = 0.84) was significantly inversely associated with GC (Huang et al., 2020). Also, findings from Linxian General Population Trial Cohort from China have indicated that meat consumption was not linked to either cardia or non‐cardia GC (Tran et al., 2005).

Several mechanisms have been proposed to explain the possible association between red meat intake and the risk of GC. Among GC risk factors, heme iron, which is abundantly contained in red meat, promotes endogenous formation of carcinogenic NOCs (Cross et al., 2003). Heme, which is known to promote the formation of endogenous NOCs in the human gastrointestinal system, would have been present in larger concentrations in red meat and processed meat (Cross et al., 2003). The increased risk of cancers such colorectal cancer and stomach cancer may be caused by NOCs from endogenous production or external nitrosamines (Lewin et al., 2006). NOCs, including nitrosamines, have been produced in the stomach by interaction between ingested nitrites or derived from nitrates with secondary and tertiary amines. Meat and fish proteins have been suggested as possible main sources for secondary and tertiary amines (Kim et al., 2002). Data have indicated that added nitrate as preservative and coloring agent is not considered carcinogenic (Kim et al., 2002). However, in the gastrointestinal tract, nitrate is converted to nitrite, so NOCs will be formed from nitrite reacting with amino substrates in food (Kim et al., 2002). NOCs are thought to increase the chance of developing GC, particularly non‐cardia GC, since they have an impact on the high levels of nitrogenous residues in the gastrointestinal system. Moreover, *H. pylori*, a dangerous and significant leading risk factor for stomach cancer, grows in part as a result of iron causing DNA damage or oxidative stress (Pérez‐Pérez & Israel, 2000; Suzuki et al., 2009). Moreover, one of the risk factors for GC may also be the way the meat is prepared, processed, and preserved. For instance, cooking meat at a high temperature will lead to the formation of heterocyclic amines and polycyclic aromatic hydrocarbons (Skog et al., 1998). Data also have indicated that high dietary salt, which is contained in cured or salted meat, damages gastric mucosa and induces significant gastric pathology and inflammation (Bergin et al., 2003). Due to a relative lack of heme iron, which is present in much greater quantities in red meat, white meat may not have the same impact as red meat (Bingham et al., 2002). Moreover, white meat is an abundant source of polyunsaturated fatty acids (PUFAs) and contains a lower level of cholesterol and saturated fat than red meat, and PUFAs are thought to prevent the development of cancer by triggering apoptosis, regulating the cell cycle and the generation of eicosanoids, and exerting an anti‐proliferative effect (Bingham et al., 2002).

Findings from the current research have indicated that the intake of different levels and frequencies of milk, yogurt, white and processed cheeses, and eggs had positive significant associations with risk of GC. However, the consumption of butter milk on a weekly basis increased risk of GC two‐folds (*p* trend <.001). The daily consumption of white cheese (OR = 4.18), processed cheese (OR = 3.25), and cooked eggs (OR = 2.44) was positively and significantly associated with the risk of GC. The current findings are consistent with the data that had reported that the consumption of dairy products significantly increased the risk of GC (OR for highest quartile = 2.7). However, after adjustment for BMI and total energy intake, dairy consumption slightly increased the risk of GC (Ward & Lopez‐Carrillo, 1999). A meta‐analysis published in 2008 found that dairy product consumption might decrease the risk of GC, but it only included case–control studies that had been conducted in China (Huang et al., 2009). The current findings are consistent with data from study in countries with different cancer risk in Latin America which had indicated that egg consumption increased the risk effect of GC when tertile 3 is compared against tertile 1 (OR = 1.7, *p* value = .021; Flores‐Luna et al., 2020). Tse and Eslick (2014) reported the presence of association between egg consumption and GI cancers, and this risk was stronger in colon cancer, with an OR of 1.29 (95% CI 1.14–1.46; *p* value heterogeneity <.22). The dose–response analysis produced similar results regardless of stratification method used. Specifically, for an intake of <3 and ≥3 eggs per week, the ORs were 1.14 (95% CI 1.07–1.22; *p* value heterogeneity = .38) and 1.25 (95% CI 1.14–1.38; *p* value heterogeneity = .25), respectively (Tse & Eslick, 2014). Also, data from two ecological studies in high risk countries, China and Brazil, had revealed that at population‐level there were geographical positive correlations between egg consumption and GC mortality (Kneller et al., 1992; Sichieri et al., 1996). Moreover, daily consumption of milk and eggs had not been associated with GC risk among males (Tokui et al., 2005). Inconsistent with the current findings, in a case–control study in Uruguay, it had been found that egg consumption was protective factor against the GC (De Stefani et al., 2004). It had been found that the larger intakes of egg and cholesterol resulted in lesser eradication of *H. pylori* by anti‐*H. pylori* treatment; however, cholesterol from eggs can increase *H. pylori* virulence through the production of cholesterol—glucosides by *H. pylori* themselves (Ikezaki et al., 2017). The current findings are also in parallel with previous data that had showed significant positive trend of increasing GC risk with increasing the frequency of cheese (RR = 3.5) and butter (RR = 1.9; Muñoz et al., 1997). However, it had been found a non‐significant association between the risk of GC and butter consumption (hazard risk (HR) = 0.37, 95% CI 0.14–1.01; Tokui et al., 2005).

The current findings have indicated that there is a significant association between weekly consumption (2–3 servings/week) of milk (OR = 2.04) and the risk of GC (*p* value of trend = .041). Consistent with the current findings, Ward and Lopez‐Carrillo (1999) reported that individual dairy products that had been associated with GC risk (highest vs. lowest quartile) were milk (more than once/day vs. less than once/week, OR = 2.2) and cheese (three or more times/week vs. less than once/month, OR = 3.8; Ward & Lopez‐Carrillo, 1999). Epidemiological studies revealed a higher risk of GC among people who consumed milk from livestock that had fed on bracken fern (Alonso‐Amelot & Avendano, 2002). Experimental animals have demonstrated that the primary chemical component, ptaquiloside, which was isolated from bracken fern, causes stomach malignancies (Gomes et al., 2012). In vitro and in a mouse model, some investigations directly demonstrated that this carcinogen might cause genetic instability and a DNA damage response in GC cells (Gomes et al., 2012). Also, it had been proposed that the immunosuppressive properties of bracken fern and their modulation of numerous physiological processes may raise the risk of developing stomach cancer (Shahin et al., 1999). However, the blood insulin‐like growth factor 1 (IGF‐1) level is elevated by milk consumption, and this had been linked to an increase in stomach cancer (Franciosi et al., 2003). Data indicated that the serum IGF‐1 levels in patients with stomach cancer might be noticeably higher than normal levels (Franciosi et al., 2003). The current findings have indicated that consumption of yogurt, drained yogurt (labneh), cooked yogurt, and ice cream had no effect on GC risk. In parallel with the present result, yogurt and cream were rarely consumed, and they were not associated with GC risk (Ward & Lopez‐Carrillo, 1999). However, the consumption of yogurt one to two times per month among men was associated with decreased risk of GC risk compared to those who ever consumed yogurt (HR = 0.69, 95% CI 0.49–0.98; Ward & Lopez‐Carrillo, 1999). Another study indicated that total dairy consumption was not associated with GC risk (OR: 1.09; 95% CI 0.96–1.25; Tian et al., 2014). Data also found that there was no significant association between cheese intake and GC risk when comparing high cheese intake versus low cheese intake (OR: 0.98; 95% CI 0.69–1.39; Tian et al., 2014).

The current study has a few limitations, starting with recall bias, which is always a concern in case–control studies, especially when evaluating dietary information. In addition, the interviewers were not blinded for the diagnosis of the participants (i.e., cases and control); nevertheless, all interviewers were well trained and treated the participants professionally and identically, regardless of their case and control status. However, this case–control study has several strengths, including the adjustment of statistical analyses for many substantial confounders is believed to strengthen our findings by eliminating the effects of these variables on GC risk. The major strengths of this study were the use of an ethnically validated FFQ; GC newly diagnosed cases and cancer‐free controls were enrolled from the major hospitals to include the different diets consumed by Jordanians; and the compliance with the questionnaire was high with an eminent response rate of >95%.

In conclusion, the consumption of milk, yogurt, some cheeses, cooked red meats, cooked chicken, and cooked eggs was significantly higher among GC group as compared to control group. The consumption of varied amounts and regularities of white cheese, processed cheese, chicken, and egg showed an increased risk of GC. The adjusted ORs showed that the risk of GC was positively associated with daily intake of red meat and weekly intakes of milk and buttermilk.

## AUTHOR CONTRIBUTIONS

**Reema F. Tayyem:** Conceptualization (lead); formal analysis (lead); methodology (equal); project administration (equal); resources (lead); supervision (lead); writing – original draft (lead); writing – review and editing (lead). **Hala Nawaiseh:** Writing – original draft (lead); writing – review and editing (equal). **Narmeen Al‐Awwad:** Project administration (equal); writing – review and editing (equal). **Tareq Al‐Jaberi:** Conceptualization (equal); data curation (equal); methodology (equal); supervision (equal); writing – review and editing (equal). **Ahmad Hushki:** Data curation (equal); supervision (equal); writing – review and editing (equal). **Sabika Allehdan:** Data curation (lead); formal analysis (lead); writing – original draft (equal); writing – review and editing (equal).

## CONFLICT OF INTEREST STATEMENT

The authors declare that they have no conflict of interest.

Placeholder Text

## Data Availability

The data that support the findings of this study are available on request from the corresponding author.
